# Neuroanatomical correlates of psychopathology in antipsychotic-naïve schizophrenia

**DOI:** 10.4103/0019-5545.58892

**Published:** 2010

**Authors:** Ganesan Venkatasubramanian

**Affiliations:** Department of Psychiatry, National Institute of Mental Health and Neurosciences, Bangalore - 560 029, India

**Keywords:** Psychopathology, schizophrenia, voxel-based morphometry

## Abstract

**Background:**

Previous Magnetic Resonance Imaging (MRI) studies using manual techniques reporting significant relationship between psychopathology and gray matter volume in schizophrenia are limited by various confounding factors. None used automated image analysis to examine gray matter volume correlates of psychopathology in antipsychotic-naïve schizophrenia patients.

**Aim:**

This study aimed at examining the relationship between psychopathology and gray matter volume abnormalities in antipsychotic-naïve schizophrenia patients.

**Patients and Methods:**

MRI of 30 antipsychotic-naïve schizophrenia (DSM-IV) patients and 27 age-, sex- education- and handedness-matched healthy controls were compared for gray matter volume differences using Optimized Voxel-based Morphometry (VBM)-an automated, rapid and unbiased technique. Psychopathology was measured using Positive and Negative Syndrome Scale (PANSS) with good inter-rater reliability. The correlations between PANSS scores and gray matter volume were examined using VBM.

**Results:**

Schizophrenia patients had significant gray matter volume deficits in frontal, cingulate, temporal, insula and precuneus cortices; thalamus, caudate and cerebellum. Positive syndrome score had significant negative correlation with left superior temporal gyrus volume. Negative syndrome score had significant inverse correlation with frontal, cingulate and cerebellar gray matter volumes.

**Conclusions:**

Cortical and cerebellar gray matter volume deficits and their significant negative correlations with psychopathology scores are supportive of ‘Cognitive Dysmetria’ in schizophrenia.

## INTRODUCTION

Schizophrenia is a complex and heterogeneous psychotic disorder with a wide range of symptoms including delusions, hallucinations, formal thought disorder, altered affect and cognitive functioning.[1] Studies over the past many years have established brain abnormalities in schizophrenia. Recent MRI studies have extended the area of interest from the gray matter of the medial temporal lobe to cortical gray matter in general. Collectively, these recent findings are compelling and suggest there is a 5-8% global reduction in cortical gray matter volume in schizophrenia.[2]

Review of published MRI studies show brain structural abnormalities in schizophrenia to involve cortical, subcortical and cerebellar regions.[3–6] No specific group of regions has yet emerged as the ‘schizophrenia circuit’, but a consensus is developing on some of the nodes that may be involved. These nodes include various sub-regions within the frontal cortex, the anterior cingulate gyrus, the thalamus, several temporal lobe sub-regions[1] and cerebellum.[7,8] These cortical, thalamic and cerebellar abnormalities in schizophrenia are explained by the unifying concept of ‘Cognitive Dysmetria’.[8]

Since brain structural abnormalities have been established in schizophrenia, parallel studies are attempted at examining the relationship of these abnormalities with psychopathology. Nonetheless, compared to the large number of studies on brain structural abnormalities in schizophrenia, there is a paucity of studies examining the neuroanatomical correlates of psychopathology in this disorder.[9]

Smaller posterior superior temporal cortex has been reported in schizophrenia patients with formal thought disorder.[10,11] Studies have demonstrated association between superior temporal cortex[12] to hallucinations in schizophrenia. Significant negative correlation between prefrontal volume and negative syndrome has also been reported.[13]

However, these MRI studies were limited in resolution of studied MR images, as well as restricted to the analysis of single predefined regions of interest.[9] In all these studies, the most prominent method used to investigate structural brain abnormalities is region-of-interest (ROI) analysis. Such analyses identify *a priori* brain regions and employ manual outlining or stereological procedures to obtain volumetric measurements. These user-dependent methods could render the results biased.[14,15] Finally, manual region-of-interest techniques are laborious, which can hinder efficient processing of large cohorts.[6]

Thus, an automated, efficient whole-brain analysis to detect structural differences would provide an unbiased means of identifying regions of structural brain abnormalities.[6] Recently, investigators have employed voxel-based morphometry (VBM), a fully automated whole-brain measurement technique, to examine structural MR images of the brain.[16] By surveying the whole brain, VBM provides a non-biased measure of highly localized regions that may not be investigated in hypothesis-based studies that employ more labor-intensive ROI measurement techniques. Voxel-based morphometry for gray matter volume abnormalities involves a voxel-wise comparison of the probability of the presence of gray matter between groups of subjects. Scans are spatially normalized into the same stereotactic space and segmented into gray matter, white matter and CSF compartments. The VBM methodology has been updated and optimized[17] to reduce errors due to systematic differences in head shape, variations in segmentation, inconsistent brain stripping and errors introduced by spatial normalization (simplified and elaborate details of this method is described in the ‘Methods’ section later).

To date, only one study has employed an automated method to examine the correlates of psychopathology in schizophrenia patients.[9] However, this study was confounded by the long-term neuroleptic exposure of the patients. It is well established that treatment with neuroleptics can alter the brain structure. Thus, no previous study has examined the gray matter volume correlates of psychopathology in antipsychotic-naïve schizophrenia using a fully automated image analysis technique.

The objective of the present study was to 1) compare 30 antipsychotic-naïve Indian schizophrenia patients with 27 age-, sex-, education- and handedness-matched healthy controls for gray matter volume differences and 2) examine for correlations between regional gray matter volume and psychopathology scores. Based on the emerging consensus on ‘cognitive dysmetria’ network abnormalities in schizophrenia, the study hypotheses were: 1) schizophrenia patients would exhibit gray matter volume deficits in neocortical association areas, thalamus and cerebellum; 2) these deficient brain regions would have significant inverse relationship with positive and negative syndrome scores.

## PATIENTS AND METHODS

### Subjects

The sample consisted of 30 antipsychotic-naïve schizophrenia patients and 27 healthy comparison subjects. The schizophrenia patients were recruited from the outpatient services of the National Institute of Mental Health and Neurosciences, Bangalore, India. The healthy controls were recruited through ‘word-of-mouth’ from consenting volunteers. Age, sex and education did not differ significantly between patients and healthy comparison subjects (*P* > 0.05) [Table 1]. All subjects (patients and controls) were right-handed.[18] DSM-IV diagnosis of schizophrenia[19] was established using the Structured Clinical Interview for the DSM-IV.[20] The diagnosis was confirmed through independent clinical interview by an experienced psychiatrist. The first episode and illness duration (mean ± SD: 41.7 ± 35.9 months) as defined by report of psychotic symptoms were assessed using the Instrument for the Retrospective Assessment of Onset of Schizophrenia.[21] None of the patients were exposed to any psychotropic medications including antipsychotics before assessments.

**Table 1:** Comparison of demographic profile of schizophrenia patients and healthy controls

Variable	Patients (*n* = 30)	Controls (*n* = 27)	Statistic	*P*
Age (years)*	30.1 ± 8.3	27.4 ± 7.0	t* = 1.3	> 0.05
Years of education*	12 ± 3	13 ± 3	t* = 1.9	> 0.05
Sex (M : F)**	21 : 9	19 : 8	x^2^** = 0.001	> 0.05

* Comparison using independent samples *t*-test;

** Comparison using Chi-square test; *P* > 0.05

### Assessment of psychopathology

The psychopathology was assessed using the Positive and Negative Syndrome Scale (PANSS).[22] Inter-rater reliability for psychopathology scores was examined with another qualified psychiatrist. The ratings were simultaneous when one of the raters (in turns) examined a series of 15 patients. The inter-rater reliability was calculated using intra-class correlation coefficient. The intra-class correlation coefficients for positive syndrome, negative syndrome and general psychopathology were > 0.9 indicating excellent inter-rater reliability. The psychopathology scores (mean ± SD) were positive syndrome (22 ± 8), negative syndrome (23 ± 9) and general psychopathology (39 ± 8).

Healthy comparison subjects were screened using the 12- item General Health Questionnaire[23] and a comprehensive mental status examination. None of the healthy comparison subject had family history of psychiatric illness in their first-degree relatives. None of the subjects (Patients and HC) scored positive for alcohol use on CAGE questionnaire.[24] None used stimulant or opiate drug. No subject had history of neurological/medical disorder. All subjects signed informed written consent. The Institute's ethics committee approved the study.

### Scanning protocol

MRI was done with 1.5 Tesla Magnetom ‘vision’ scanner. T_1_- weighted three-dimensional Magnetization Prepared Rapid Acquisition Gradient Echo sequence was performed (TR = 9.7 ms, TE = 4 ms, nutation angle = 12°, FOV = 250 mm, slice thickness 1-mm, NEX = 1) yielding 160 sagittal slices.

### Image processing

The Optimized VBM protocol was implemented within Matlab 7.1 (Mathworks, Natick, Mass) through Statistical Parametric Mapping 2 (SPM2).[25,26] SPM2 uses an updated segmentation model with improved bias correction component that can segment brain abnormalities better than previous versions (Wellcome Department of Imaging Neuroscience, London; http://www.fil.ion.ucl.ac.uk/spm).

Preprocessing of structural data followed a number of defined stages:[17]

1. Creation of a separate gray and white matter templates
2. Segmentation and extraction of a brain image.
3. Normalization of gray/white matter images.
4. Segmentation and extraction of normalized whole brain images.
5. Modulation and Smoothing

### Creation of customized templates

Each structural MRI was normalized to the standard statistical parametric mapping T_1_ template; segmented into CSF, gray matter and white matter compartments; Study-specific gray and white matter templates were created by averaging all the 57 (30 schizophrenia patients and 27 healthy controls) smoothed normalized gray/white matter images. Then smoothed (8-mm full width at half maximum isotropic Gaussian kernel) and averaged to create gray and white matter templates in stereotactic space. The optimized templates were created from the whole subject group rather than a subset in order to avoid any potential bias for spatial normalization.[17]

### Segmentation and extraction of a brain image

Segmentation and extraction is a fully automated procedure to remove scalp tissue, skull and dural venous sinus voxels. The statistical parametric mapping segmentation employs a mixture model cluster analysis (after correcting for nonuniformity in image intensity) to identify voxel intensities that match particular tissue types combined with *a priori* probabilistic knowledge of the spatial distribution of tissues.[27] Initially, the original structural MRI is segmented into gray and white matter images. This is followed by various automated procedures involving erosion followed by conditional dilatation that would result in removal of unconnected non-brain voxels from the segmented images. These series of operations would yield extracted gray and white matter partitions in native space.[17]

### Normalization of gray/white matter images

Extracted gray and white matter images were spatially normalized to match the gray and white matter templates. Spatial normalization is an image-processing step, more specifically an image registration method. Human brains differ in size and shape, and one goal of spatial normalization is to deform human brain scans to match a template brain scan. After spatial normalization, a specific location in one subject's brain scan corresponds to the same location in another subject's brain scan. The steps involved in the spatial normalization are 1) specification/estimation of warp-field and 2) application of warp-field with re-sampling. Such normalization typically involves not only translation and rotation, but also scaling and nonlinear warping of the brain surface to match a standard template. In a study involving multiple subjects, spatial normalization is performed to ensure the correspondence and hence the uniformity of brain regional localizations. This would facilitate comparison and various other statistical analyses.

The normalization parameters were then reapplied to the original structural images to maximize optimal segmentation of fully normalized images, and these normalized images were re-sliced to a final voxel size of 1 mm^3^ and segmented into gray/white matter and CSF/non-CSF partitions.

### Segmentation and extraction of normalized whole brain images

The optimally normalized whole brain structural images, which are now in stereotactic space (based on Montreal Neurological Institute (MNI) template[28]), are then segmented into gray and white matter, CSF, and non-CSF partitions and subject to a second extraction of normalized segmented gray/white matter images. The brain extraction step is repeated at this stage because some non-brain voxels from scalp, skull or venous sinuses in the optimally normalized whole brain images could still remain outside the brain margins on segmented gray/white matter images.[27]

### Modulation and smoothing

As a result of nonlinear spatial normalization, the volumes of certain brain regions may grow, whereas others may shrink. In order to preserve the volume of a particular tissue (gray or white matter or CSF) within a voxel, a further processing step is incorporated. This involves multiplying (or modulating) voxel values in the segmented images by the Jacobian determinants derived from the spatial normalization step. In effect, an analysis of modulated data tests for regional differences in the absolute amount (volume) of gray matter, whereas analysis of unmodulated data tests for regional differences in concentration of gray matter (per unit volume in native space).[16]

The modulated gray matter images are then smoothed. Images are often smoothed (similar to the ‘blur’ effect used in some image-editing software) by which voxels are averaged with their neighbors, typically using a Gaussian filter with a 12 mm Full-Width Half Maximum (FWHM) Kernel, to make the data less noisy. The process of smoothing conditioned the residuals to conform more closely to the Gaussian random field model underlying the statistical process used for adjusting ‘*P*’ values.[29,30]

Thus, the Optimized VBM pre-processing yielded normalized, segmented, modulated and smoothed images (Gray Matter, White Matter and CSF images) with a voxel size of 1 mm^3^.[16,17] GM, White Matter (WM), CSF and total Intracranial Volumes (ICV) were calculated automatically using Matlab scripts and SPM.[31]

### Statistical analysis

#### Statistical analyses of clinical variable and global brain matter volumes

The statistical analysis was performed using the Statistical Package for Social Sciences - version 10.0.1. The clinical data were analysed using the independent samples *t*-test and Chi-square test. The global gray matter, white matter and CSF volume differences between patients and healthy controls were analysed using the analysis of covariance (ANCOVA) with the intracranial volume as a covariate.

### Statistical parametric mapping: Optimized voxel-based morphometry analyses

#### Group comparison for regional gray matter volume differences

Group comparisons for regional gray matter volume differences were performed using ‘single subject: Conditions and covariates’ analysis within the framework of general linear model in SPM2 with intracranial volume as the confounding covariate and age and sex as nuisance covariates. Statistical parametric maps were constructed to test for regional gray matter volume differences between patients and controls.

These were automatically analyzed by the SPM software on a voxel-by-voxel basis. Significance corrections for multiple comparisons over whole brain were done using false discovery rate (FDR) correction (*P* < 0.05).[32] False discovery rate (FDR) is a new approach to the multiple comparisons problem. Instead of controlling the chance of any false positives (as in Bonferroni or random field methods), FDR controls the expected proportion of false positives among supra-threshold voxels. An FDR threshold is determined from the observed *P*-value distribution and hence is adaptive to the observations in a specific dataset.

### Correlations between gray matter regions and positive and negative syndrome scale scores

Statistical parametric maps were also examined for correlation between specific gray matter volumes and positive and negative syndrome scores (corrected for total PANSS score). The general psychopathology score was not utilized for correlational analysis because of its heterogeneity. These specific regions of gray matter volume deficits were identified from the results of the group comparison analysis as per above [Table 2]. This analysis was examined for correlates in specific *a priori* regions rather than an exploratory whole brain analysis. Since the analysis was pre-selected gray matter voxels (that were chosen based on the results of regional gray matter volume deficits obtained after group comparison analysis as described above), significance was inferred with uncorrected *P* values.

**Table 2:** Regions of gray matter volume deficits in antipsychotic-naïve schizophrenia patients (*n* = 30) in comparison to matched healthy subjects (*n* = 27)

Brain region	BA	Coordinates*			*T*	P**
		
		*x*	*y*	*z*
Left superior frontal gyrus	8	−2	43	46	2.3	<0.001
Right superior frontal gyrus	6	6	8	49	4.5	<0.001
Left inferior frontal gyrus	47	−30	14	−21	3.1	<0.001
Right inferior frontal gyrus	46	44	38	13	4.2	<0.001
Left cingulate gyrus	24	−8	−4	37	5.1	<0.001
Right cingulate gyrus	24	8	4	46	6.1	<0.001
Left superior temporal gyrus	38	−34	14	−23	3.7	<0.001
Right superior temporal gyrus	39	46	−54	10	3.5	<0.001
Left insula	22	−40	−23	3	4.9	<0.001
Right insula	13	42	−16	−3	5.2	<0.001
Left precuneus	31	−12	−59	27	4.2	<0.001
Left thalamus (Medio-dorsal Nucleus)	-	−10	−15	10	4.1	<0.001
Left caudate	-	−12	−3	15	3.9	<0.001
Left cerebellum posterior declive	-	−6	−82	−13	4.1	<0.001

BA - Brodmann area

* Talairach and Tournoux coordinates of peak difference

** All scores significant (*P* < 0.05) after false discovery rate correction for multiple comparisons over the whole brain

### Localization of gray matter region

The coordinates of significant voxels were converted from Montreal Neurological Institute space to Talairach and Tournoux coordinates[33] using a nonlinear transform approach.[34] Using the Talairach and Tournoux coordinates, the brain regions were localized utilizing an automated software.[35]

## RESULTS

Schizophrenia patients did not significantly differ from healthy comparison subjects in age, sex and years of education (*P* > 0.05). The PANSS scores (Mean ± SD) of schizophrenia patients were as follows: Positive syndrome (22 ± 8), negative syndrome (23 ± 9) and general psychopathology (39 ± 8). Schizophrenia patients (1412 ± 134 mL) and healthy controls (1409 ± 107 mL) did not differ significantly in ICV (*t* = 0.09; *P* = 0.9). Patients had significantly smaller gray matter volume than healthy controls after controlling for ICV. After controlling for ICV, CSF volume was significantly greater in patients than healthy controls whereas WM volume did not differ significantly between patients and healthy controls [Table 3].

**Table 3:** Comparison for global brain matter volume (mL) differences between antipsychotic-naïve schizophrenia patients (n = 30) and healthy controls (n = 27)

Variable	Patients (Mean ± SD)	Controls (Mean ± SD)	F*	‘*P*’
Gray matter volume	632 ± 56	661 ± 45	21.3	< 0.001
White matter volume	393 ± 52	403 ± 47	3.3	0.07
Cerebrospinal fluid volume	388 ± 51	346 ± 40	21.4	< 0.001

* Analysis of covariance with intracranial volume as a covariate; df = 2,54.

### Regional gray matter volume differences between patients and healthy controls

**Figure 1:**
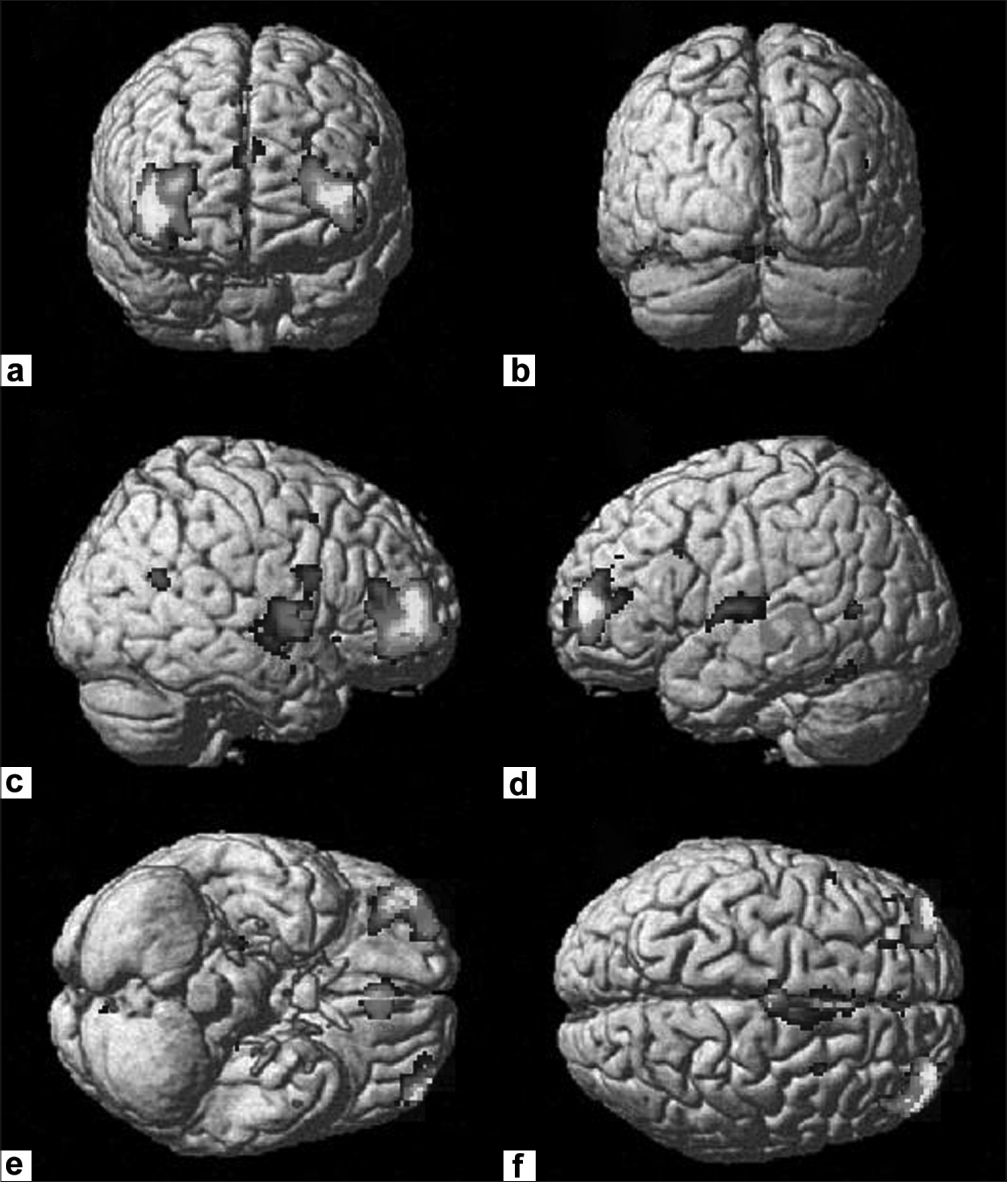
Rendered images depicting the gray matter volume deficits in schizophrenia patients (n = 30) in comparison to healthy controls (n = 27). The deficit regions are highlighted in yellow and red - Representative of regions in Table 2.

**Figure 2:**
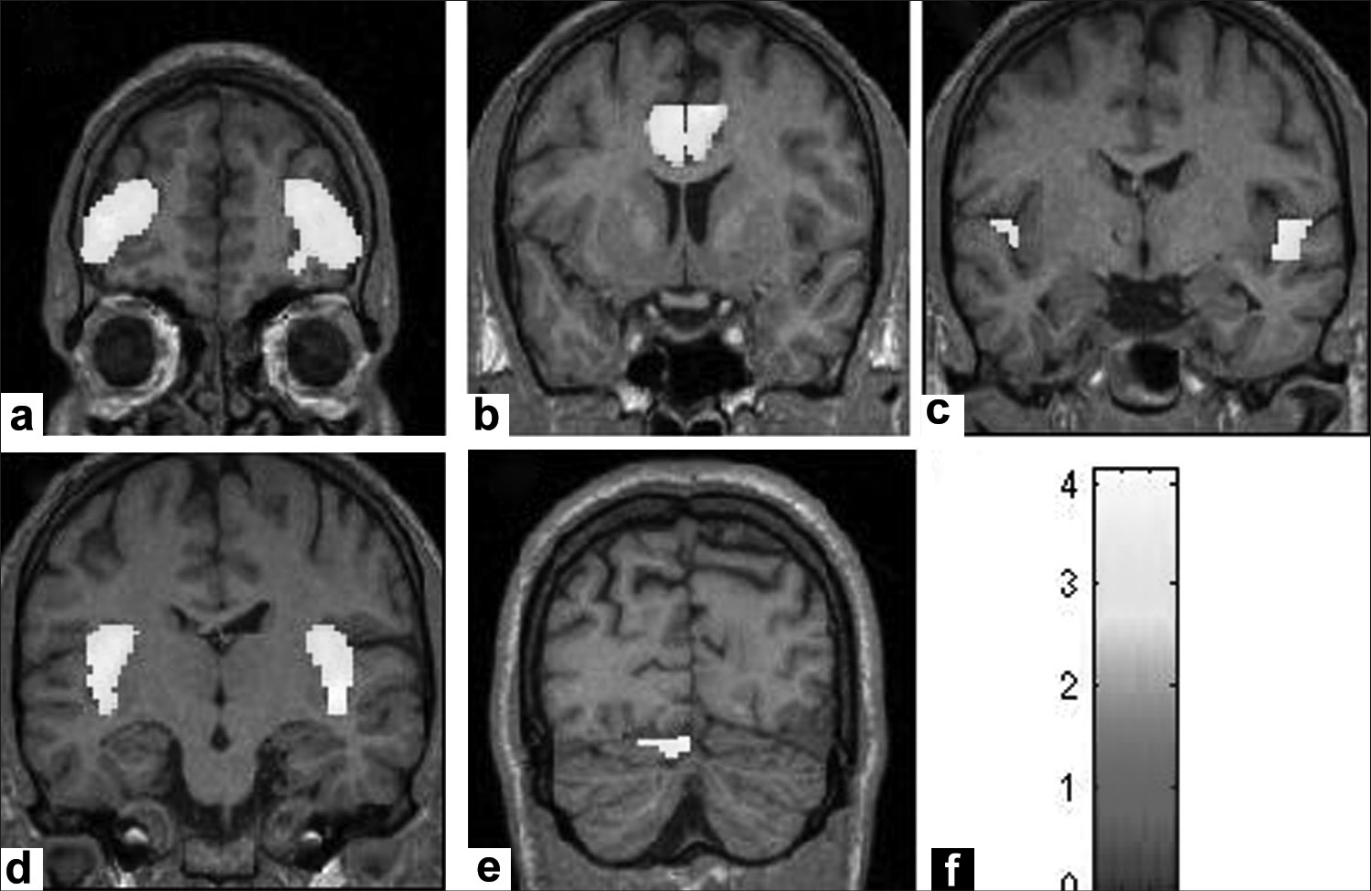
Figure shows gray matter volume deficits in schizophrenia patients (n = 30) in comparison to healthy controls (n = 27). The deficient regions are highlighted in yellow. The regional deficits are as follows: (a) Frontal gyri, (b) Cingulate gyri, (c) Temporal gyri, (d) Insula and (e) Cerebellum. The color bar (f) is representative of the ‘T’ scores given in Table 2.

**Figure 3:**
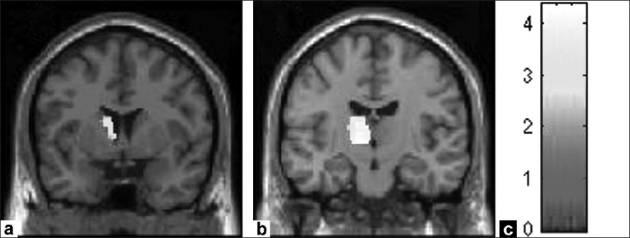
Figure shows gray matter volume deficits in schizophrenia patients (n = 30) in comparison to healthy controls (n = 27). The deficient regions are highlighted in yellow. The regional deficits are as follows: (a) Left caudate nucleus, (b) Left thalamus. The color bar (c) is representative of the ‘T’ scores given in Table 2.

The optimized voxel-based morphometric analysis revealed significant gray matter volume deficits in schizophrenia patients in comparison to healthy controls in the following regions: Frontal, cingulate, temporal, insula and precuneus cortices; thalamus, caudate nucleus and cerebellum [Table 2; Figures 1–3].

### Correlations between gray matter regions and positive and negative syndrome scale scores

**Figure 4:**
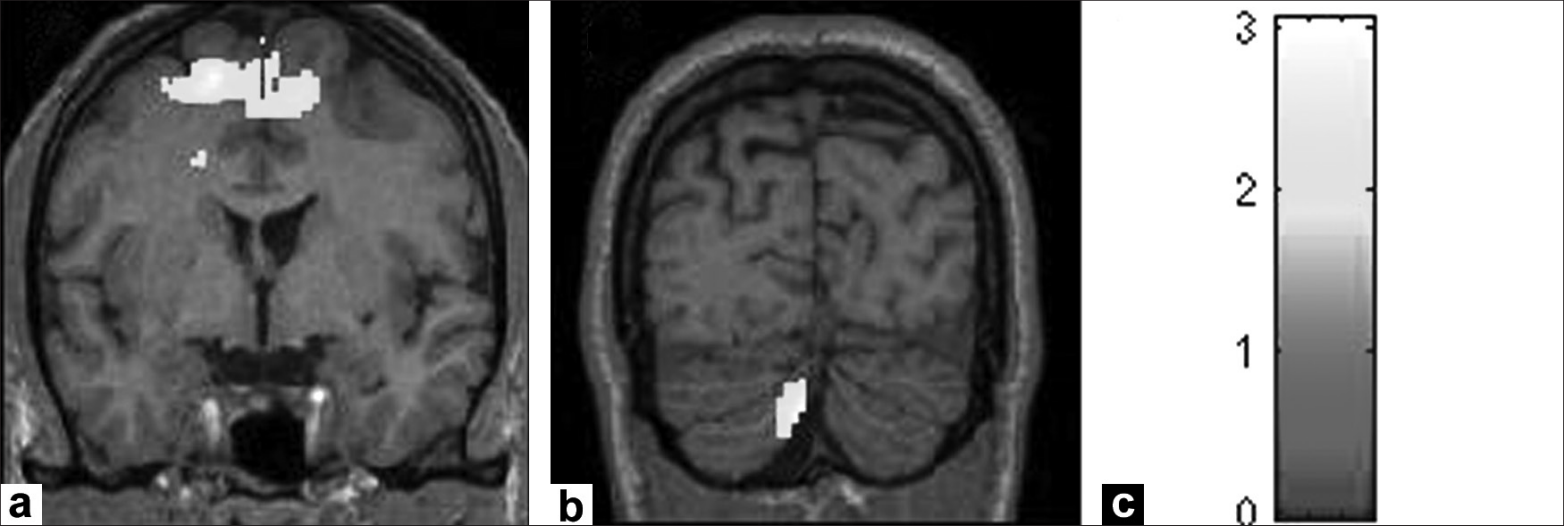
Figure shows regional gray matter volumes (highlighted in yellow) that had significantly inverse relationship with negative syndrome score in schizophrenia patients (n = 30); (a) Frontal gyri, (b) Cerebellum. The color bar (c) is representative of the ‘T’ scores given in Table 4.

Significant negative correlation was found between positive syndrome score and left superior temporal gyrus (Talairach and Tournoux peak correlation coordinates: *×* = −38, *y* = 22, *z* = −30; *T* = 2.1; *P* = 0.025). The negative syndrome score had a significantly inverse correlation with frontal and cingulate cortices as well as cerebellum [Table 4; Figure 4].

**Table 4:** Gray matter regions having significant inverse correlation with negative syndrome in schizophrenia patients

Brain region	BA	Coordinates*			*T* score	‘*P*’
		
		*x*	*y*	*z*
Left superior frontal gyrus	6	-2	-3	63	2.0	0.03
Right superior frontal gyrus	8	20	45	47	2.4	0.01
Left middle frontal gyrus	6	-48	12	49	2.8	0.005
Right middle frontal gyrus	8	42	28	47	2.4	0.01
Left cingulate gyrus	32	-2	8	42	2.1	0.02
Right cingulate gyrus	32	2	8	42	2.3	0.01
Left cerebellum	-	-6	-82	-13	2.2	0.02

BA - Brodmann area

* Talairach and Tournoux coordinates of peak difference

## DISCUSSION

This is the first study to examine antipsychotic-naïve schizophrenia patients for gray matter volume correlates of psychopathology using a completely automated, rapid and unbiased technique namely the Optimized Voxel-based Morphometry (VBM). In this study, antipsychotic-naïve schizophrenia patients showed significant gray matter volume deficits in frontal, cingulate, temporal, insula and precuneus cortices; thalamus, caudate nucleus and cerebellum in comparison with age-, sex-, education- and handedness-matched healthy controls. In schizophrenia patients, positive syndrome score had a significantly negative correlation with left superior temporal gyrus; whereas the negative syndrome score had a significantly inverse correlation with frontal and cingulate cortices as well as cerebellum.

### Gray matter volume deficits

The frontal cortical abnormalities demonstrated in this study are in tune with previous observations using manual region-of-interest based studies.[36–38] The gray matter volume deficits in caudate, cerebellum, frontal and temporal lobes support earlier ROI studies in antipsychotic-naïve schizophrenia.[39] Thalamic volume deficit, especially involving the medio-dorsal nucleus is supportive of earlier observations.[31,40] Also, this study could replicate previous ROI-based finding of caudate and cerebellar volume deficits in Indian antipsychotic-naïve schizophrenia patients.[41] The study findings are comparable with the previous VBM studies on antipsychotic-naïve schizophrenia patients from the western countries.[42–44] In addition, the study findings are in tune with an earlier VBM study on antipsychotic-naïve schizophrenia patients from India, which examined an independent cohort of patients of relatively smaller sample size.[45] Thus, the gray matter volume deficits in schizophrenia patients identified in this study involved cortical, subcortical as well as cerebellar regions.

From a neuroanatomical systems perspective, a recent comprehensive review of literature suggested that major brain areas and circuits in the brain that have been implicated in the connections and functions of the dorsal prefrontal cortex are affected in schizophrenia.[5] Specifically, this involved the cortico-cortical and cortico-thalamo-cerebellar brain regions. The present study findings are in tune with this view.

The prefrontal cortex is the region rostral to the motor and premotor cortices, receiving the cortical projection of the mediodorsal thalamic nucleus (MD), and is distinguished by a granular layer IV.[5] The prefrontal cortex receives its main specific thalamic input from the thalamic mediodorsal nucleus (MD) with a separate input from the pulvinar. The MD projection forms a major part of the anterior thalamic radiation and the inferior thalamic peduncle,[5] a projection that is distributed to all areas of the prefrontal cortex - namely the frontal as well as the cingulate gyri. The frontal, cingulate and thalamic volume deficits, as demonstrated in this study, are suggestive of abnormalities in the fronto-thalamic networks in schizophrenia. In this study, along with these fronto-thalamic abnormalities, deficits were observed in caudate and cerebellar gray matter volumes also. This is in tune with the neuropathological abnormalities involving cortical, basal ganglia and thalamic brain regions in schizophrenia.[46] Interestingly, cortical, thalamic and cerebellar abnormalities in schizophrenia are explained by the unifying concept of ‘Cognitive Dysmetria’.[8]

### Neuroanatomical correlates of psychopathology

In this study, the positive syndrome score correlated significantly and negatively with left superior temporal gyral gray matter volume. This is in tune with previous observations in medicated schizophrenia patients.[9,47–49] Dysfunction to HG could impair ‘bottom-up’ processing, giving greater perceptual control to ‘top-down’ mechanisms. Such cognitive functioning is associated with the generation of some of the positive symptoms like hallucinations.[50,51]

On the other hand, negative syndrome score had a significant inverse relationship with frontal and cingulate cortices as well as cerebellum. These findings are in tune with previous manual region-of-interest-based studies on chronic, medicated, schizophrenia patients.[52] The integrity of prefrontal cortex is vital for attention, working memory, motivation, volition and various related executive functions.[53] Hence, impairment of prefrontal function might potentially underlie the pathophysiology of various features of negative syndrome like amotivation, avolition, inattention and working memory deficits. Interestingly, the frontal and cerebellar correlates of negative syndrome offer further support to the concept of ‘Cognitive Dysmetria’ in schizophrenia.[8]

### Study findings support cognitive dysmetria in schizophrenia

The concept of ‘Cognitive Dysmetria’ proposes schizophrenia to be a ‘misconnection syndrome’ of neural circuitry involving cortical, thalamic and cerebellar brain regions.[8] It is suggested that the disruption of cortical-thalamic-cerebellar-cortical circuit (CCTCC) leads to impairment in synchrony, or the smooth coordination of mental processes. When synchrony is impaired, the patient suffers from a cognitive dysmetria, and the impairment in this basic cognitive process defines the phenotype of schizophrenia and produces its diversity of symptoms.[8] The cortical areas implicated in this circuit involve primarily prefrontal cortex and various important association cortices namely the superior temporal, cingulate and precuneus cortices.[8] In this study, gray matter volume deficits were observed in schizophrenia in brain regions underlying the CCTCC circuit. Importantly, many of these regions had significantly inverse relationship with the psychopathology of schizophrenia implying lesser the volume more severe the symptoms of schizophrenia. Together, these observations offer further support to the concept of ‘cognitive dysmetria’ in schizophrenia.

### Methodological issues

This is the first study to examine antipsychotic-naïve schizophrenia patients for neuroanatomical correlates of psychopathology using a completely automated image analysis technique - namely the Optimized Voxel-based morphometry (VBM). The optimized VBM technique has several methodological advantages.[17] The optimization steps, by excluding non-brain voxels before normalization and subsequent segmentation, avoid the potential bias due to systematic variations in skull size and shape or scalp thickness. Utilizing study-specific templates give greater sensitivity to detect neuroanatomical correlates. The potential confound of ventricular abnormalities influencing GM analysis was avoided by performing spatial normalization based only on segmented GM.[17]

Some of the other methodological advantages of the study include the following: 1) antipsychotic-naïve status of the patients during the assessments, 2) SCID interview to establish the diagnosis of the patients, 3) independent confirmation of the diagnosis by an experienced psychiatrist, 4) excellent inter-rater reliability for PANSS ratings, 5) age-, sex-, education-, handedness matched controls and 6) use of 1-mm MRI slices with no inter-slice gap.

## CONCLUSIONS

This is the first study to examine antipsychotic-naïve schizophrenia patients for gray matter volume correlates of psychopathology using a completely automated, rapid and unbiased technique namely the Optimized Voxel-Based Morphometry. In this study, antipsychotic-naïve schizophrenia patients showed significant gray matter volume deficits in frontal, cingulate, temporal, insula and precuneus cortices; thalamus, caudate nucleus and cerebellum. In schizophrenia patients, positive syndrome score had a significantly negative correlation with left superior temporal gyrus; whereas the negative syndrome score had a significantly inverse correlation with frontal and cingulate cortices as well as cerebellum. Together, these findings support the concept of ‘Cognitive Dysmetria’ in schizophrenia.

## References

[1] Schultz SK, Andreasen NC (1999). Schizophrenia. Lancet.

[2] Lewis S, Keshavan MS, Murray RM (1997). Psychopathology and brain dysfunction: Structural-imaging studies. Neurodevelopment and adult psychopathology.

[3] Wright IC, Rabe-Hesketh S, Woodruff PW, David AS, Murray RM, Bullmore ET (2000). Meta-analysis of regional brain volumes in schizophrenia. Am J Psychiatry.

[4] Shenton ME, Dickey CC, Frumin M, McCarley RW (2001). A review of MRI findings in schizophrenia. Schizophr Res.

[5] Fallon JH, Opole IO, Potkin SG (2003). The neuroanatomy of schizohprenia: Circuitry and neurotransmitter systems. Clin Neurosci Res.

[6] Honea R, Crow TJ, Passingham D, Mackay CE (2005). Regional deficits in brain volume in schizophrenia: A meta-analysis of voxel-based morphometry studies. Am J Psychiatry.

[7] Andreasen NC, Paradiso S, O'Leary DS (1998). “Cognitive Dysmetria” as an integrative theory of schizophrenia: A dysfunction in cortical-subcortical-cerebellar circuitry?. Schizophr Bull.

[8] Andreasen NC (1999). A unitary model of schizophrenia: Bleuler's “fragmented phrene” as schizencephaly. Arch Gen Psychiatry.

[9] Gaser C, Nenadic I, Volz HP, Buchel C, Sauer H (2004). Neuroanatomy of “hearing voices”: A frontotemporal brain structural abnormality associated with auditory hallucinations in schizophrenia. Cereb Cortex.

[10] Shenton ME, Kikinis R, Jolesz FA, Pollak SD, LeMay M, Wible CG (1992). Abnormalities of the left temporal lobe and thought disorder in schizophrenia: A quantitative magnetic resonance imaging study. N Engl J Med.

[11] Menon RR, Barta PE, Aylward EH, Richards SS, Vaughn DD, Tien AY (1995). Posterior superior temporal gyrus in schizophrenia: Grey matter changes and clinical correlates. Schizophr Res.

[12] Barta PE, Pearlson GD, Powers RE, Richards SS, Tune LE (1990). Auditory hallucinations and smaller superior temporal gyral volume in schizophrenia. Am J Psychiatry.

[13] Gur RE, Cowell PE, Latshaw A, Turetsky BI, Grossman RI, Arnold SE (2000). Reduced dorsal and orbital prefrontal gray matter volumes in schizophrenia. Arch Gen Psychiatry.

[14] Wolkin A, Rusinek H, Vaid G, Arena L, Lafargue T, Sanfilipo M (1998). Structural magnetic resonance image averaging in schizophrenia. Am J Psychiatry.

[15] Kubicki M, Shenton ME, Salisbury DF, Hirayasu Y, Kasai K, Kikinis R (2002). Voxel-based morphometric analysis of gray matter in first episode schizophrenia. Neuroimage.

[16] Ashburner J, Friston KJ (2000). Voxel-based morphometry: The methods. Neuroimage.

[17] Good CD, Johnsrude I, Ashburner J, Henson RN, Friston KJ, Frackowiak RS (2001). A voxel-based morphometric study of ageing in 465 normal adult human brains. Neuroimage.

[18] Annett M (1967). The binomial distribution of right, mixed and left handedness. Quarterly J Exp Psychol.

[19] American Psychiatric Association (1994). DSM-IV: Diagnostic and Statistical Manual of Mental Disorders.

[20] First MB, Spitzer RL, Gibbon M, Williams JB (1997). Structured Clinical Interview for DSM-IV Axis I Disorders.

[21] Hafner H, Riecher-Rossler A, Hambrecht M, Maurer K, Meissner S, Schmidtke A (1992). IRAOS: An instrument for the retrospective assessment of onset of schizophrenia. Schizophr Res.

[22] Kay SR, Fizbein A, Opler A (1987). The positive and negative syndrome scale for schizophrenia. Schizophr Bull.

[23] Goldberg DP, Gater R, Sartorius N (1997). The validity of two versions of the GHQ in the WHO study of mental illness in general health care. Psychol Med.

[24] Ewing JA (1984). Detecting alcoholism: The CAGE Questionnaire. JAMA.

[25] Friston K, Ashburner J, Frith CD, Poline JB, Heather JD, Frackowiak RS (1995a). Spatial registration and normalization of images. Hum Brain Mapp.

[26] Friston K, Holmes AP, Worsley K, Poline JB, Frith CD, Frackowiak RS (1995b). Statistical parametric maps in functional imaging: A general linear approach. Hum Brain Mapp.

[27] Ashburner J, Friston KJ (1999). Nonlinear spatial normalization using basis functions. Hum Brain Mapp.

[28] Evans AC, Collins DL, Mills SR (1993). 3D statistical neuroanatomical models from 305 MRI volumes. IEEE Nucl Sci Symp Medical Imaging Conference Proc.

[29] Ashburner J, Neelin P, Collins DL, Evans A, Friston K (1997). Incorporating prior knowledge into image registration. Neuroimage.

[30] Worsley KJ, Marrett S, Neelin P, Vandal AC, Friston KJ, Evans AC (1996). A unified statistical approach for determining significant signals in images of cerebral activation. Hum Brain Mapp.

[31] Ananth H, Popescu I, Critchley HD, Good CD, Frackowiak RS, Dolan RJ (2002). Cortical and subcortical gray matter abnormalities in schizophrenia determined through structural magnetic resonance imaging with optimized volumetric voxel-based morphometry. Am J Psychiatry.

[32] Genovese CR, Lazar NA, Nichols TE (2002). Thresholding of statistical maps in functional neuroimaging using the false discovery rate. Neuroimage.

[33] Talairach J, Tournoux P (1998). A coplanar stereotaxic atlas of a human brain.

[34] Brett M, Johnsrude IS, Owen AM (2002). The problem of functional localization in the human brain. Nat Rev Neurosci.

[35] Lancaster JL, Woldorff MG, Parsons LM, Liotti M, Freitas CS, Rainey L (2000). Automated Talairach atlas labels for functional brain mapping. Hum Brain Mapp.

[36] Hirayasu Y, Tanaka S, Shenton ME, Salisbury DF, DeSantis MA, Levitt JJ (2001). Prefrontal gray matter volume reduction in first episode schizophrenia. Cereb Cortex.

[37] Yamasue H, Iwanami A, Hirayasu Y, Yamada H, Abe O, Kuroki N (2004). Localized volume reduction in prefrontal, temporolimbic, and paralimbic regions in schizophrenia: An MRI parcellation study. Psychiatry Res.

[38] Wible CG, Anderson J, Shenton ME, Kricun A, Hirayasu Y, Tanaka S (2001). Prefrontal cortex, negative symptoms, and schizophrenia: An MRI study. Psychiatry Res.

[39] Cahn W, Hulshoff Pol HE, Bongers M, Schnack HG, Mandl RC, van Haren NE (2002). Brain morphology in antipsychotic-naïve schizophrenia: A study of multiple brain structures. Br J Psychiatry.

[40] Gilbert AR, Rosenberg DR, Harenski K, Spencer S, Sweeney JA, Keshavan MS (2001). Thalamic volumes in patients with first-episode schizophrenia. Am J Psychiatry.

[41] Venkatasubramanian G, Gangadhar BN, Jayakumar PN, Janakiramaiah N, Keshavan MS (2003). Striato-cerebellar abnormalities in never-treated schizophrenia: Evidence for neurodevelopmental etiopathogenesis. German J Psychiatry.

[42] Job DE, Whalley HC, McConnell S, Glabus M, Johnstone EC, Lawrie SM (2002). Structural gray matter differences between first-episode schizophrenics and normal controls using voxel-based morphometry. Neuroimage.

[43] Kubicki M, Shenton ME, Salisbury DF, Hirayasu Y, Kasai K, Kikinis R (2002). Voxel-based morphometric analysis of gray matter in first episode schizophrenia. Neuroimage.

[44] Salgado-Pineda P, Baeza I, Perez-Gomez M, Vendrell P, Junque C, Bargallo N (2003). Sustained attention impairment correlates to gray matter decreases in first episode neuroleptic-naive schizophrenic patients. Neuroimage.

[45] Jayakumar PN, Venkatasubramanian G, Gangadhar BN, Janakiramaiah N, Keshavan MS (2005). Optimized voxel-based morphometry of gray matter volume in first-episode, antipsychotic-naive schizophrenia. Prog Neuropsychopharmacol Biol Psychiatry.

[46] Heckers S (1997). Neuropathology of schizophrenia: Cortex, thalamus, basal ganglia, and neurotransmitter-specific projection systems. Schizophr Bull.

[47] Levitan C, Ward PB, Catts SV (1999). Superior temporal gyral volumes and laterality correlates of auditory hallucinations in schizophrenia. Biol Psychiatry.

[48] Rajarethinam RP, DeQuardo JR, Nalepa R, Tandon R (2000). Superior temporal gyrus in schizophrenia: A volumetric magnetic resonance imaging study. Schizophr Res.

[49] Sumich A, Chitnis XA, Fannon DG, O'Ceallaigh S, Doku VC, Faldrowicz A (2005). Unreality symptoms and volumetric measures of Heschl's gyrus and planum temporal in first-episode psychosis. Biol Psychiatry.

[50] Aleman A, Bocker KB, Hijman R, de Haan EH, Kahn RS (2003). Cognitive basis of hallucinations in schizophrenia: Role of top-down information processing. Schizophr Res.

[51] Antrobus J (1991). Dreaming: Cognitive processes during cortical activation and high afferent thresholds. Psychol Rev.

[52] Wassink TH, Andreasen NC, Nopoulos P, Flaum M (1999). Cerebellar morphology as a predictor of symptom and psychosocial outcome in schizophrenia. Biol Psychiatry.

[53] Fuster JM (2001). The prefrontal cortex-an update: Time is of essence. Neuron.

